# A Combined Open and Endovascular Approach for the Treatment of a Torcular Dural Arteriovenous Fistula

**DOI:** 10.7759/cureus.1874

**Published:** 2017-11-24

**Authors:** Luke Tomycz, Ali S Haider, Jefferson Miley

**Affiliations:** 1 Pediatric Neurosurgery, Dell Children's Medical Center of Central Texas; 2 Texas A&M College of Medicine; 3 Neurointerventional Surgery, Seton Brain and Spine Institute

**Keywords:** cerebrovascular, dural arteriovenous fistula, endovascular neurosurgery, pediatric, torcula

## Abstract

Cranial dural arteriovenous fistulae (dAVFs) are complex vascular lesions, rarely encountered within the pediatric population. Endovascular embolization has revolutionized the management of these lesions and should be regarded as the first-line treatment for the majority of dAVFs; however, in a subset of particularly complicated lesions, traditional routes of access either do not exist or have been eliminated in the course of prior embolization attempts. We describe the case of an 11-year-old boy with a complicated torcular dAVF refractory to multiple attempts at standard endovascular treatment. He was cured after direct surgical puncture of the superior sagittal sinus to obtain vascular access for coil embolization of the venous sac. We review the technical aspects of this particular case while highlighting the anatomic features of those dAVFs that require nontraditional, surgical access for appropriate treatment. We also describe a simple algorithm, which may help in identifying the small subset of dAVF that requires a hybrid open and endovascular approach to effectively access the fistulous point and achieve disconnection.

## Introduction

Cranial dural arteriovenous fistulae (dAVFs) are complex vascular lesions, rarely encountered within the pediatric population. In neonates, dural AVFs may lead to life-threatening congestive failure, prompting urgent treatment, while older children with dAVFs typically present with signs and symptoms related to venous hypertension [1]. Endovascular embolization has revolutionized the management of these lesions and should be regarded as the first-line treatment for the majority of dAVFs; however, in a subset of particularly complicated lesions, traditional routes of access either do not exist or have been eliminated in the course of prior embolization attempts [2]. Here, we describe the case of a young boy with a persistent torcular dAVF after multiple, prior trans-arterial embolizations who was ultimately cured via a combined open and endovascular approach. We review the technical aspects of this particular case while highlighting the anatomic features of those dAVFs that require nontraditional, surgical access for appropriate treatment.

## Case presentation

**Figure 1:**
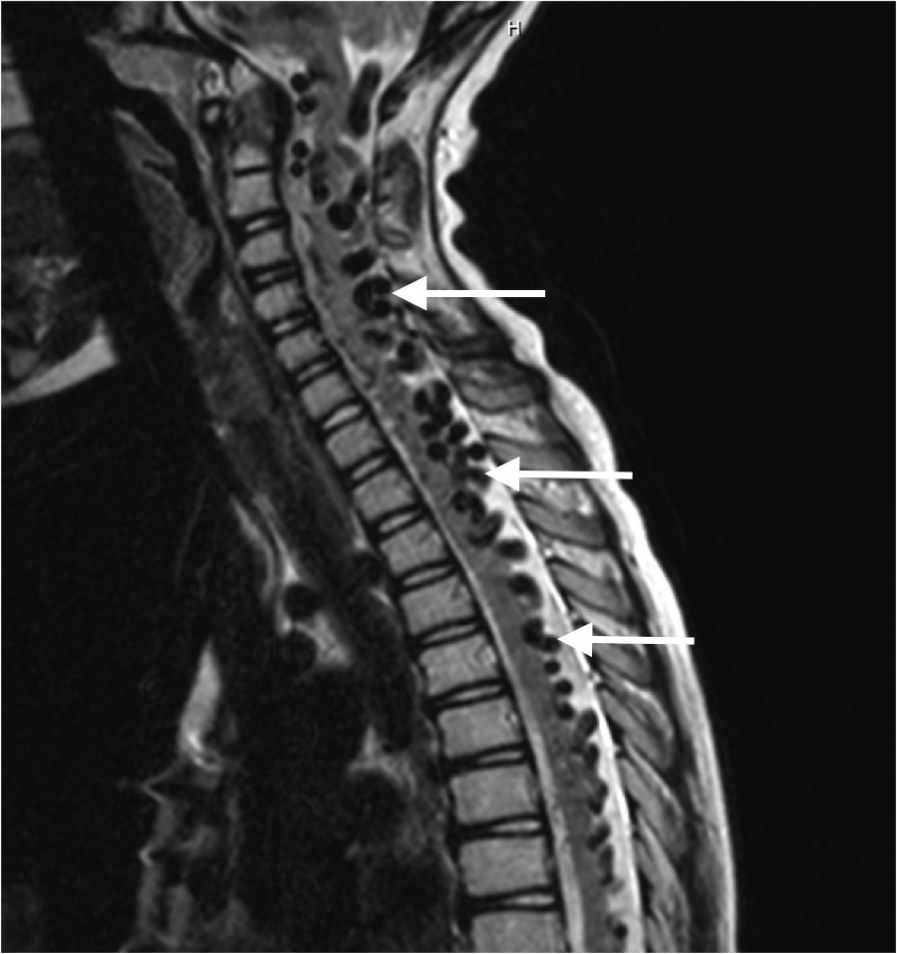
Sagittal magnetic resonance imaging showing serpiginous veins (white arrows) throughout the cervicothoracic spine

An 11-year-old male was referred to us for increasing frequency of seizures, occurring multiple times daily, despite several antiepileptic medications. His clinical picture was further complicated by constant, debilitating headaches, generalized fatigue, and significant cognitive delay. Surveillance magnetic resonance imaging (MRI) revealed the progressive dilation of serpiginous venous varices within the cervical spine (Figure 1). 

**Figure 2:**
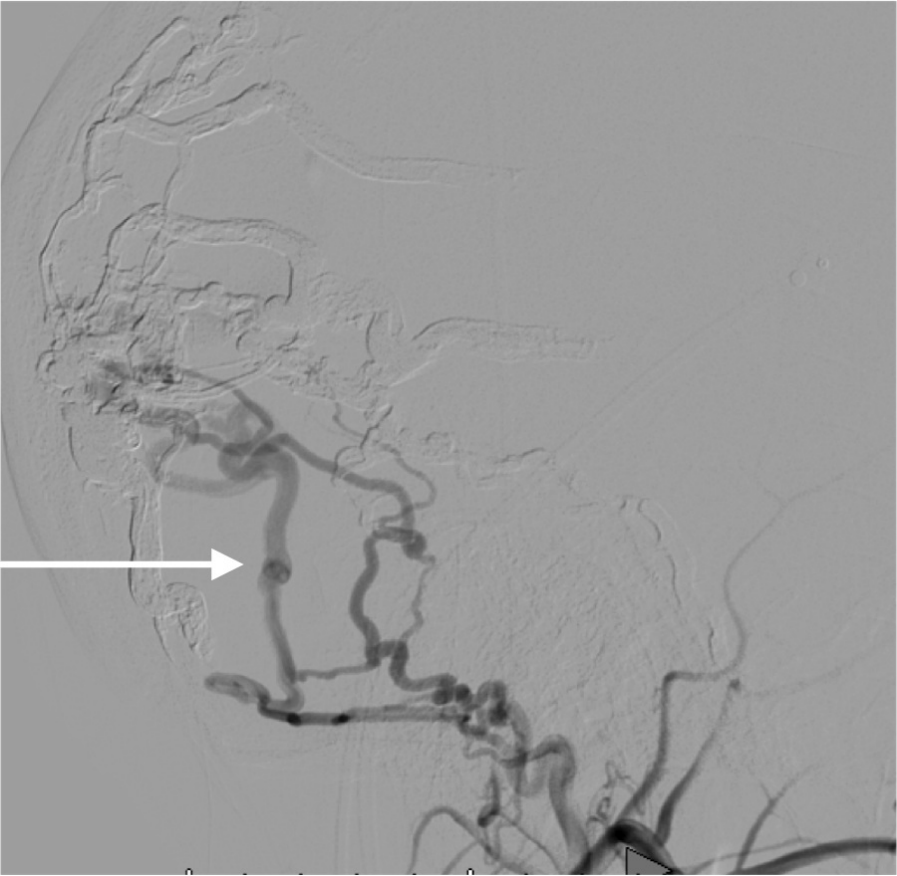
Left lateral external carotid artery angiogram (arrow), early arterial phase

**Figure 3:**
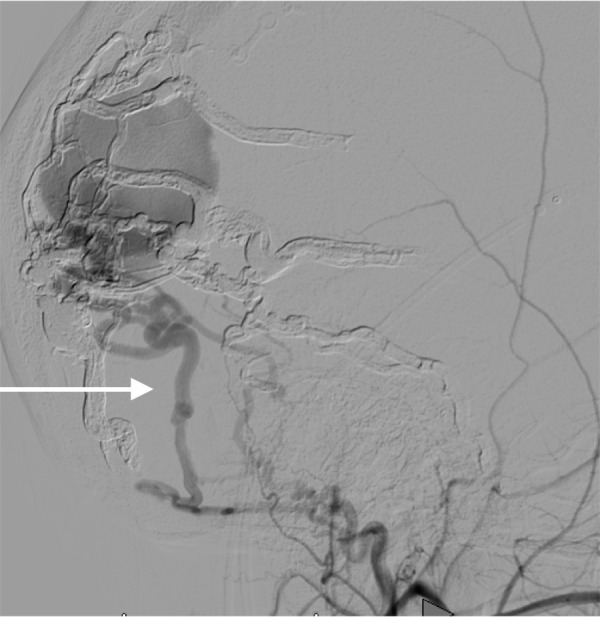
Left lateral external carotid artery angiogram (arrow), late arterial phase

Most of these findings were felt to be attributable to a known, partially treated torcular dAVF. This boy had undergone multiple prior embolizations, the last of which was complicated by a total loss of vision in the right eye. A cerebral angiogram revealed a persistent, torcular dAVF with multiple feeders from the bilateral external carotid arteries as well as the meningeal branches of the left internal carotid artery (Figure 2, Figure 3)

**Figure 4:**
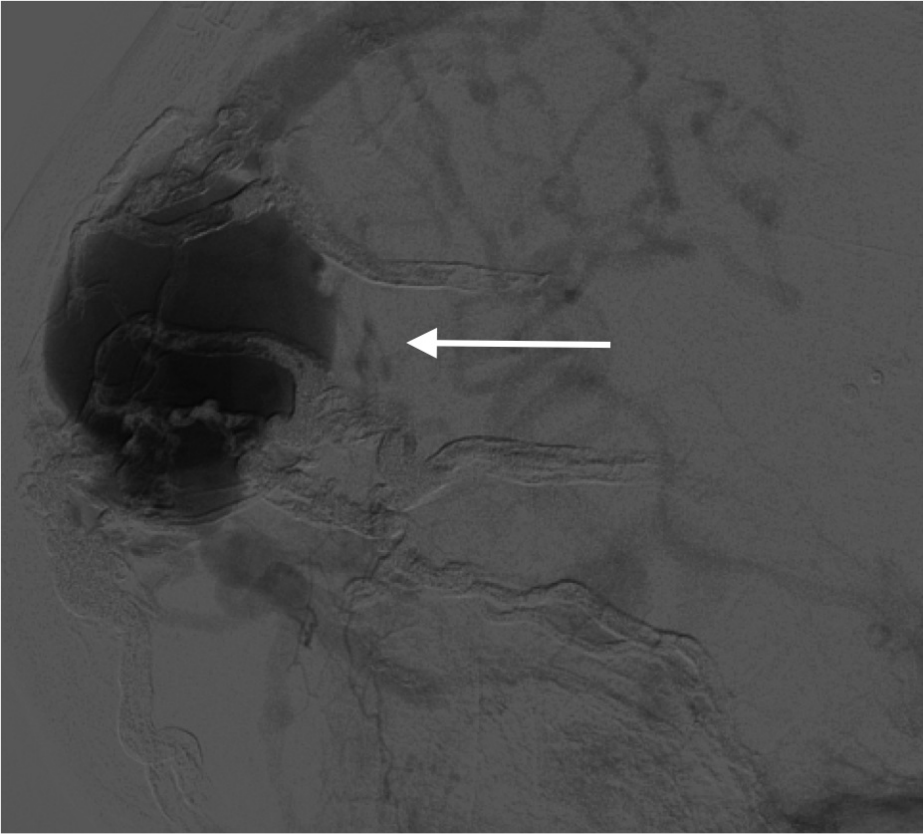
Left lateral external carotid artery angiogram (arrow), venous phase

There was also noted to be complete occlusion of the transverse and sigmoid sinuses, as well as the internal jugular veins, bilaterally, and the superior sagittal sinus filled in a retrograde fashion before refluxing into numerous cortical veins (Figure 4).

Operation

**Figure 5:**
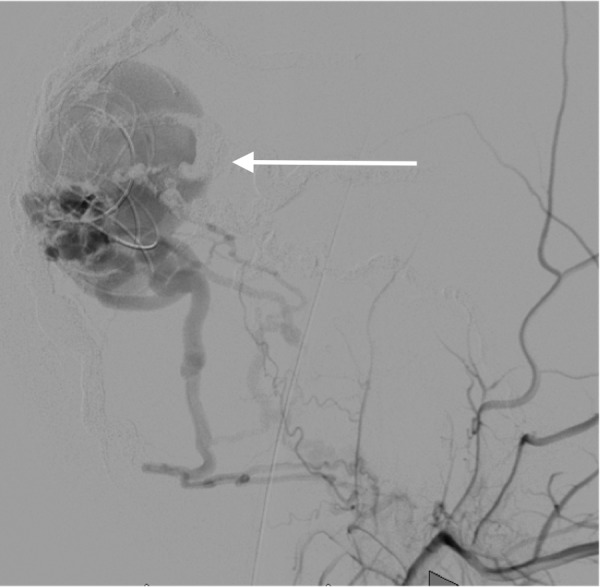
Left external carotid artery angiogram (arrow) at different stages of the coiling procedures

**Figure 6:**
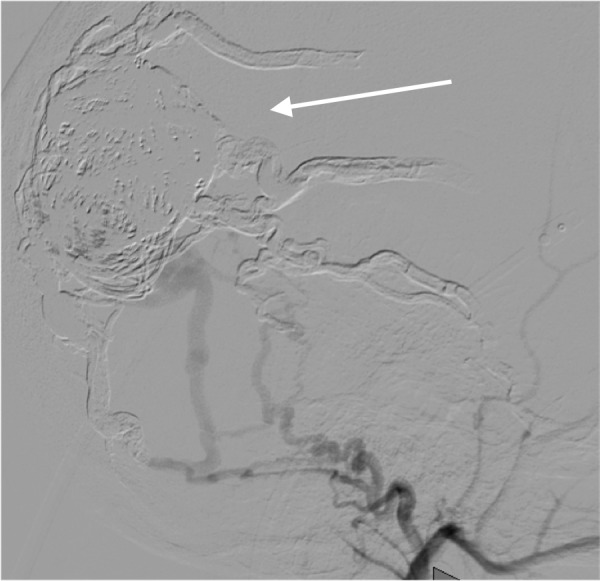
Left external carotid artery angiogram at different stages of the coiling procedures

After registering the head with neuronavigational technology, we planned a small craniotomy over the posterior third of the superior sagittal sinus. We then directly punctured the sinus with a 5French (5F) needle and upon seeing the flow of bright red blood, advanced a wire under fluoroscopic guidance that appeared to follow the convexity of the skull along the course of the sinus before entering the massively dilated torcula. The needle was then withdrawn, and a 5F dilator was inserted over the wire and stitched in place to the adjacent dura. Contrast was manually injected, and we obtained confirmation that the tip of the dilator was located in the venous sac. The dilator was then connected to a continuous, pressurized saline flush to maintain patency. The patient’s wound was irrigated and closed with staples, and the patient was transported to the biplane angiography suite. A standard 5F diagnostic sheath was placed in the right femoral artery, and an angled, tapered catheter and 0.27 glidewire were used to access the left external carotid artery (ECA) for control runs. We then connected long, intravenous (IV) extension tubing to the end of the dilator that was exiting the cranial incision. We introduced a microcatheter and 0.14 micro guidewire under fluoroscopic guidance, positioning the tip of the catheter in the massive venous sac of the fistula. Twenty-five hundred units of heparin were given at this time as a bolus. Microcatheter injection along with a control angiogram confirmed appropriate positioning, and we proceeded to embolize the venous sac with over 35 large coils (Penumbra Inc., California, US), monitoring progress with serial trans-arterial ECA angiograms (Figure 5, Figure 6).

After the final coil was placed, ECA angiography showed near complete obliteration of the fistulous connection, and the femoral sheath was withdrawn and pressure was held. The patient was transferred back to the operating room for removal of the 5F dilator from within the superior sagittal sinus under direct visualization. Bleeding from the sinus was addressed with a figure-eight stitch (4.0 Nurolon, Ethicon Inc., New Jersey, US).

Postoperative Course

**Figure 7:**
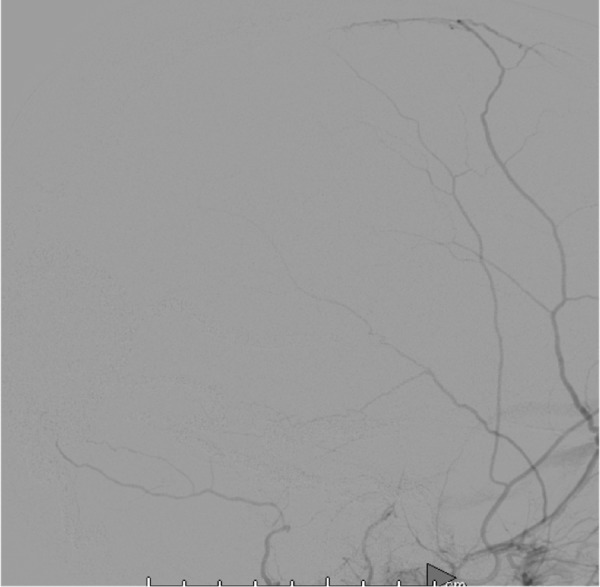
Three-month post-operative angiogram showing total obliteration of the fistula, left external carotid artery lateral view

**Figure 8:**
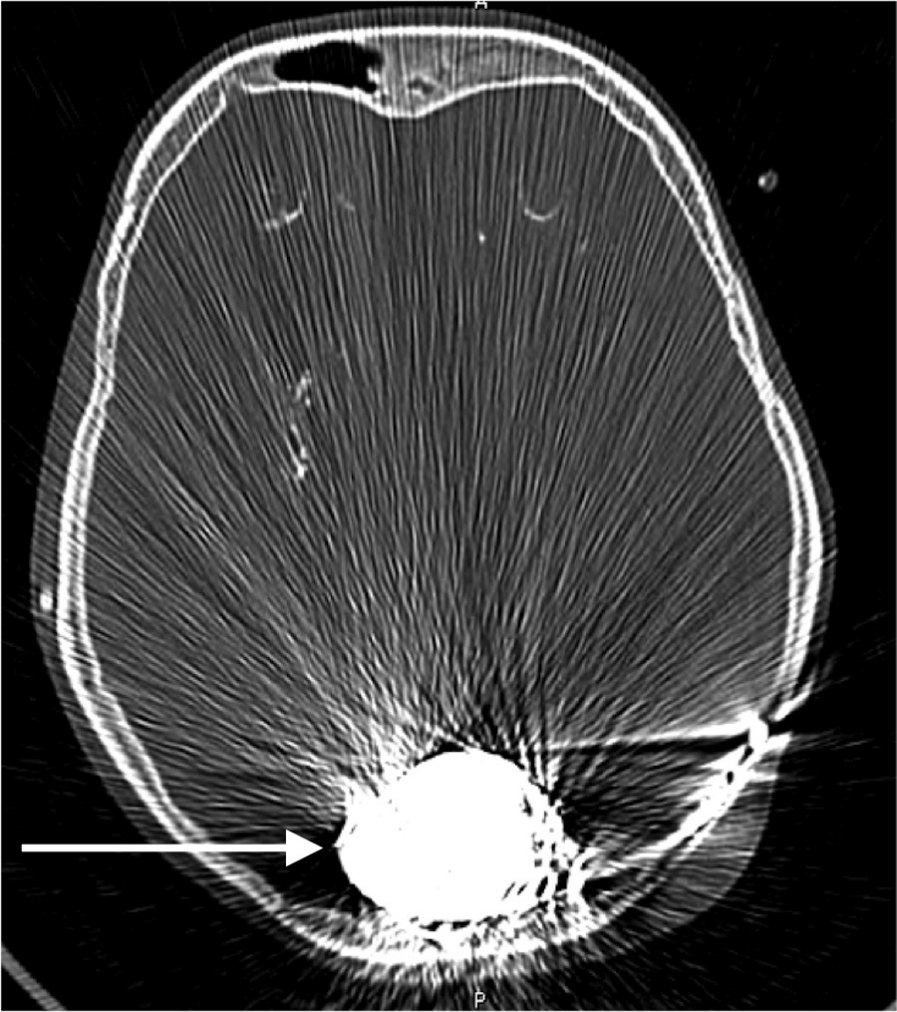
Post-operative computed tomography showing large coil mass (arrow)

There were no immediate post-operative complications. The patient was watched overnight in the intensive care unit and went home the following day. The three-month follow-up angiogram confirmed obliteration of the fistula with preservation of flow within the superior sagittal sinus (Figure 7, Figure 8).

Clinically, treatment led to the near-cessation of seizures on antiepileptic drugs, dramatic alleviation of headaches, and marked improvement in energy level and school performance.

## Discussion

**Figure 9:**
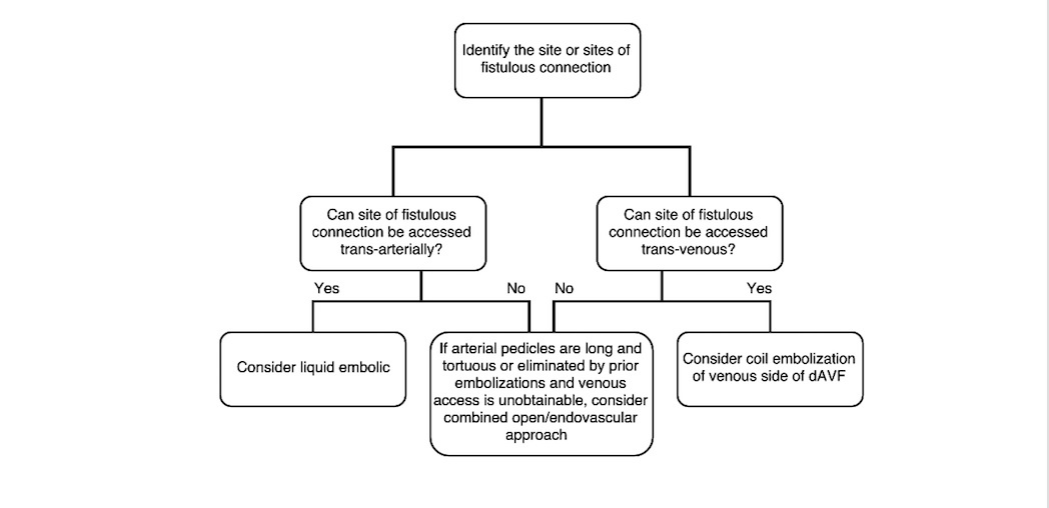
Simple algorithm for determining whether a combined open/endovascular approach should be considered in the treatment of dural arteriovenous fistulae

While the vast majority of dAVFs can be treated via endovascular methods, a small subset of especially complicated lesions may require creative, alternative approaches to access and close off the fistulous connection. In the patient described earlier, arterial access was limited by both by the embolization of feeders from prior interventions as well as the profound tortuosity of existing feeders. Venous access was also limited, in this case, due to bilateral occlusion of the transverse and sigmoid sinuses as well as both internal jugular veins. Open surgical disconnection of the fistula would have been technically challenging and associated with significant risk of morbidity. Therefore, a hybrid approach using endovascular coiling after obtaining access via open surgical methods was felt to be the optimal treatment strategy. Radiosurgery is also a treatment option for difficult dAVFs or those refractory to other modes of therapy, although the clinical effect is characterized by a delay of up to a year. In this case, the fistulous point involved several, small dural arteries dumping into a large, dilated venous confluence and, so, the actual target for disconnection was not discrete. The long-term effects of radiation therapy need to be carefully considered, especially in the pediatric population [3]. Creative approaches may sometimes be used to gain access to the fistulous point for effective disconnection. For example, various authors have described techniques for direct access to the middle meningeal artery or various transcranial approaches to puncture the superior sagittal sinus or the traverse-sigmoid sinus [4-5]. The cut-down of the superior orbital vein has been used for the treatment of indirect fistulas of the cavernous sinus when other routes are unavailable [6-7]. Prior grading schemes (e.g. Borden, Cognard) have provided guidance regarding which dAVFs should be recommended for treatment with a focus on cortical venous reflux as a sign of an aggressive fistula. We propose a simple algorithm that can be followed to identify the rare subset of malformations that are unlikely to be cured with traditional methods and may mandate a combined open-endovascular approach (Figure 9).

After it has been determined that treatment is indicated, the fistulous point must carefully be identified, and this often requires a painstaking and thorough review of the images. Once the fistulous point or points have been identified, the trans-arterial and trans-venous routes of access are carefully considered. If an arterial pedicle is long and extremely tortuous, it may not be a good candidate artery to effectively disconnect the fistula. Having said this, the use of liquid embolic materials and balloon-mounted catheters have allowed embolization to be effectively carried out over increasingly large distances [8-10]. Dangerous potential anastomoses need to be kept in mind and assiduously looked for, and may present a contraindication to proceeding with an embolization. The other, and many would argue more important, side of the equation when it comes to dAVF is the venous side. Even in a situation where a balloon-mounted catheter can be used and liquid embolic can be pushed through a tortuous arterial pedicle, the embolic agent must be able to reach and obliterate the venous side of the fistula, effectively disconnecting all other feeders from the venous side. In the case of this child, the massive venous sac which was the point of fistulous connection would have been virtually impossible to obliterate with liquid embolic alone and, thus, it was felt that coils needed to be used to effectively fill and thrombose the venous side of the fistula. During angiography, runs should be held out late into the venous phase to study the venous outflow of a dAVF. Computed tomography or magnetic resonance venography can also be helpful in determining the potency of major venous routes (e.g., internal jugular vein and major cranial sinuses) to the point of fistulous connection. After these considerations have been made, and if no standard arterial or venous access routes are available or if the venous side of the fistula involves massive venous aneurysms, open surgical methods for access should be entertained. This will spare the patient the morbidity associated with additional and likely ineffectual embolizations, as in the patient considered herein who underwent countless embolizations and suffered from right-sided blindness as a complication of embolization as well as continued epilepsy and cognitive decline from the persistence of his untreated fistula.

## Conclusions

We described the case of an 11-year-old boy with a complicated torcular dAVF refractory to multiple attempts of standard endovascular treatment. He was cured after direct surgical puncture of the superior sagittal sinus to obtain vascular access for coil embolization of the venous sac. We described an algorithm that may help in identifying the small subset of dAVF that requires a hybrid open and endovascular approach to effectively access the fistulous point and achieve disconnection.
